# The influence of cofactors on allergic reactions to food

**DOI:** 10.1186/2045-7022-5-S3-P51

**Published:** 2015-03-30

**Authors:** Astrid Versluis, Harmieke Van Os-Medendorp, Astrid Kruizinga, Anouska Michelsen, Marty Blom, Geert Houben, André Knulst

**Affiliations:** 1 University Medical Centre Utrecht, Utrecht, The Netherlands; 2 TNO, Zeist, The Netherlands

## Background

Cofactors, like exercise, alcohol consumption and use of several types of medication, may influence the occurrence and severity of allergic reactions to food. However there is limited evidence on how often cofactors play a role and what their influence is on allergic reactions.

## Objective

To get insight in the influence of cofactors on allergic reactions to food.

## Methods

A baseline questionnaire was completed by every adult patient (≥18 years) visiting the outpatients department Allergology for the first time. Patients with food allergy documented by typical allergic symptoms to food and a positive skinpricktest, ImmunoCAP or food challenge were included. Outcome measures were the reported suspected influence of the co-factors exercise, alcohol and medication on allergic complaints to food.

## Results

502 patients were included between 2003-2011. Most patients had allergy to several types of food (mean: 4 different foods). Most common food allergies were fruit and vegetables (73%), nuts (57%) and peanuts (38%). Prevalence of other atopic diseases were asthma 64%, hay fever 61% and atopic dermatitis 75%. Of all patients, 5% used antacids and 2% used NSAIDs. Beta blockers, angiotensin-receptor inhibitors, ACE inhibitors, proton-pomp inhibitors and H2-receptor antagonists were used by ≤0.6% of the patients. Only 13% of all patients indicated to experience influence of one of the cofactors on the severity of allergic complaints; exercise was reported by 9%, alcohol consumption by 5% and use of analgetics by 0.6%.

## Conclusion

Only a small percentage of patients used medication that might influence the occurrence and severity of allergic reactions to food. Exercise and alcohol were the most frequently reported cofactors but only in less than 10% of the patients. The importance of these cofactors on severity and eliciting dose for individual patients remains to be elucidated, but at the population level, the influence of these cofactors on threshold distributions is likely to be small.

